# Family care of people with severe mental disorders: an integrative review

**DOI:** 10.1590/0104-1169.0138.2562

**Published:** 2015

**Authors:** Maria Juan-Porcar, Lledó Guillamón-Gimeno, Azucena Pedraz-Marcos, Ana María Palmar-Santos

**Affiliations:** 1 Doctoral student, Departament d'Infermeria, Facultat de Ciències de la Salut, Universitat Jaume I, Castelló, Spain. Associate Professor, Departament d'Infermeria, Universitat Jaume I, Castelló, Spain; 2 MSc, Associate Professor, Departament d'Infermeria, Universitat Jaume I, Castelló, Spain; 3 PhD, Full Professor, Sección Departamenta de Enfermería, Universidad Autónoma de Madrid, Madrid, Spain

**Keywords:** Nursing, Caregivers, Mental Disorders, Review

## Abstract

**OBJECTIVE:**

to analyze the scientific literature on home-based family care of people with severe mental illness.

**METHOD:**

integrative review of 14 databases (CINALH, Cochrane Plus, Cuidatge, CUIDEN, Eric, IBECS, EMI, ISOC, JBI COnNECT, LILACS, PsycINFO, PubMed, SciELO, and Scopus) searched with the key words "family caregivers", "severe mental illness", and "home" between 2003 and 2013.

**RESULTS:**

of 787 articles retrieved, only 85 met the inclusion criteria. The articles appeared in 61 journals from different areas and disciplines, mainly from nursing (36%). The countries producing the most scientific literature on nursing were Brazil, the UK, and the US, and authorship predominantly belonged to university centers. A total of 54.12% of the studies presented quantitative designs, with descriptive ones standing out. Work overload, subjective perspectives, and resources were the main topics of these papers.

**CONCLUSIONS:**

the international scientific literature on home-based, informal family care of people with severe mental disorder is limited. Nursing research stands out in this field. The prevalent topics coincide with the evolution of the mental health system. The expansion of the scientific approach to family care is promoted to create evidence-based guidelines for family caregivers and for the clinical practice of professional caregivers.

## Introduction

Mental illnesses or disorders can be classified into two major groups: common mental disorders (CMDs) and severe mental illness (SMI)^(^ 1 ^-^ 3 ^)^. CMDs are more frequent and less disabling for individuals and are usually treated by a single health care professional^(^ 1 ^)^. SMIs are more disabling and meet three conditions: (a) a medical diagnosis that includes a psychotic disorder (excluding organic) or certain personality disorder, b) a disease and treatment duration greater than two years, and, (c) the presence of disability, understood as moderate or severe difficulty with overall functioning (work, social, and family)^(^ 4 ^)^. Examples of SMIs include schizophrenia, bipolar disorder, delusional disorder, and schizoaffective disorder.

Epidemiological data on the prevalence of SMI in the general population are difficult to obtain because of variability among information sources. However, the international scientific community agreed that between 2.5 and 3% of the adult population presents an SMI^(^ 1 ^)^. The global disease burden from disability attributable to mental, neurological and substance use disorders reaches 14%^(^ 5 ^)^. The economic cost of mental disorders in countries with a market economy is close to 3% of the GDP^(^ 6 ^)^. The cost of mental disorders in the European Union is estimated to range between 3 and 4% of the GDP^(^ 7 ^)^.

During the history of humankind, the societal treatment of individuals with SMI has included mainly imprisonment in institutions, such as nursing homes or mental asylums. This trend was reversed during the second half of the twentieth century, a period in international history that witnessed great changes that enabled the integration of people with SMI into society. According to the World Health Organization (WHO), these changes included the discovery of new drugs that permitted novel social interventions, the rise of movements defending human rights, and the incorporation of mental and social components into the definition of mental health^(^ 8 ^)^. This so-called psychiatric reform set aside the old model of asylum care and emphasized a new model centered on community mental health care^(^ 9 ^-^ 11 ^)^. Furthermore, the development of primary care driven by WHO in the Declaration of Alma-Ata^(12) ^gained renewed strength.

Gradually, people with SMI have been integrated into society, meaning that the responsibilities of caring for them have been transferred from institutions to the community^(^ 13 ^)^. Estimates indicate that between 40 and 90% of people who suffer from mental problems live with or retain close contact with relatives^(^ 14 ^)^. This new model of community mental health care entails shared care of the person with SMI. The agents in charge of this care are health care professionals (formal care) and family caregivers (informal care). Families take an active role in caring for sick family members, making it a workable and unavoidable remedy in the community context^(^ 15 ^-^ 17 ^)^. 

A European study^(^ 18 ^)^, which included 442 caregivers of people with SMI, offers information on caregivers' profiles, revealing the following data: caregivers are primarily women (73-88%), the mean age of caregivers is 51-66 years, 21-84% of them live with the sick family member, 25% work outside the home, 48-61% provide care for longer than 10 years, and 13-48% devote a minimum of 31 hours a week to care.

Changes from the model of asylum mental health care to the community care model have impacts on the service provided. People with SMI coexist mostly with their family; thus, health professionals and family members take care of them. Formal and informal shared care is a key point in the positive development of the individual in the community; for this reason, primary health care professionals need informal caregivers. The mental health nursing professional cares for the individual with SMI with the help of the family caregiver. Therefore, we ask ourselves the following question: what do we know regarding home-based family care for people with SMI? This is a reality that must be more carefully examined, and such an examination is the purpose of the present study.

## Method

Using a qualitative approach, the present study analyzed the scientific literature during the last decade on the home-based family care of people with SMI. The implementation of the study involved an integrative review of the literature using a process of systematizing and analyzing results from independent studies aimed at understanding a particular topic^(^ 19 ^)^. 

The following steps are required to complete such a review (there are small variations among different authors)^(^ 19 ^-^ 22 ^)^: the selection of the research question, definition of inclusion and exclusion criteria, categorization of selected studies, critical analysis of findings through the identification of differences and conflicts, interpretation of findings, and information synthesis.

The selection of the question resulted from the need to determine the scientific literature on the home-based family care of people with SMI. The research was conducted in February and March 2013, as part of a broader study on the home-based family care of people with SMI.

The strategy for article identification and selection consisted of searching for scientific articles indexed in databases (DBs) from several fields of scientific knowledge (nursing, psychiatry, psychology, and education) in sources that were both statewide (Cuidatge, CUIDEN, IME and ISOC) and international (CINALH, Cochrane, Eric, IBECS, JBI COnNECT, LILACS, PsycINFO and PubMed, SciELO, Scopus).

The search strategy was based on the appearance of the key words "family caregivers," "severe mental illness", and "home" in the title or abstract in searches based on natural language (Spanish and English) and controlled vocabulary (Health Sciences Descriptors, (DeCS) and Medical Subject Headings (MeSH). The searches were limited to the years 2003-2013 and the following languages: Spanish, English, and Portuguese. To meet the inclusion criteria, articles had to contain the three combinations of key words, investigate a population of legal age (at least 18 years of age), and be written in any of the three languages listed. Articles that did not meet the above criteria were excluded.

**Figure 1 -:**
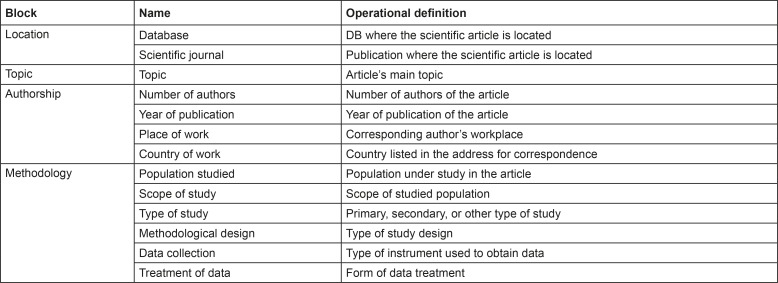
Relationship of variables investigated in scientific articles. Castelló de la Plana, Spain, 2013

Once consensus was reached regarding the relevant information from each article, researchers synthesized the information. The research variables and an operational definition of the information gathered are presented in Figure 1. The variables are categorical/qualitative and polytomous^(^ 23 ^)^.

The articles were selected according to the inclusion criteria, and the research variables were defined through peer reviews. Next, the information was entered into a spreadsheet created specifically for the purpose. Discrepancies among the reviewers were resolved by consensus. The gathered data were summarized in the form of a frequency distribution and treated statistically using a computer spreadsheet.

## Results

The search strategy located 787 articles. Only 85 (10.80%) fulfilled the inclusion criteria. The article distribution by DB is presented in Table 1. Of the 85 articles selected, only one (1.18%) appeared in three DBs, eight (9.41%) were found in two DBs, and 76 (89.41%) occurred in only a single DB.

**Table 1 -:** Location of articles in the consulted DBs. Castelló de la Plana, Spain, 2013

Database	Detected articles	Selected articles	%
PubMed	381	26	30.59
Scopus	124	14	16.47
Cochrane Plus	11	9	10.59
IBECS	35	9	10.59
SciELO	27	9	10.59
LILACS	69	8	9.41
CINAHL	39	5	5.88
CUIDEN	40	2	2.35
JBI COnNECT	2	2	2.35
Cuidatge	37	1	1.17
ISOC	1	0	0
IME	0	0	0
PsycINFO	10	0	0
Eric	11	0	0
TOTAL	787	85	100%

The DBs with the greatest precision and specificity regarding the search strategy and inclusion criteria were JBI COnNECT and Cochrane Plus, with 100 and 81.81%, respectively, of detected articles selected. Overall, 33% of the articles detected by SciELO were selected, followed by IBECS with 25.71%, CINAHL (12.82%), Scopus (11.29%), LILACS (12%), PubMed (6.82%), CUIDEN (5%), and Cuidatge (2.7%). 

The selected articles were found in 61 scientific journals covering different areas (health, social, economic, and educational areas) and disciplines (nursing, medicine, psychology, and sociology). There were 22 (36%) nursing journals. Brazil with eight journals was the country with the largest number, followed by Great Britain with four and the US with three journals. 

In all, nine topics related to family caregivers were identified based on the contents of the articles. These topics are presented in Table 2.

**Table 2 -:** Main topics in articles. FC represents family caregiver. Castelló de la Plana, Spain, 2013

Main topic	Topics included (number of items)
Working overload 25 (29.5%)	Objective overload (15)
	Subjective overload (2)
	Assessment instruments (5)
	Economic aspects (2)
	Mental health models and impact on caregivers (1)
Subjective perspectives 15 (17.6%)	FC experiences (1)
	FC perceptions (5)
	Coping (2)
	Perceived needs and shortcomings of the caregiver (2)
	Needs of the caregiver assessed with instrument (1)
	Caregiver expectations (2)
	Use requirements or standards of mental health services (1)
	FC perspective (1)
Resources 12 (14.1%)	New technologies (4)
	Instruments/scales that help FC (1)
	Program for SMI acute phase care (1)
	Psychoeducational programs (2)
	Breathing devices (3)
	Support groups (1)
Formal/informal caregiver relationship 8 (9.4%)	FC as case manager (1)
	FC as resource evaluator (3)
	FC as formal caregiver’s informant (1)
	FC as recipient of care (2)
	FC and his or her perception of participation in the care (1)
Quality of life (8.2%)	7 Articles
FC profile (7.1%)	6 Articles
Sociocultural aspects (5.9%)	5 Articles
Health prevention/care (5.9%)	5 Articles
Overprotection (2.3%)	2 Articles

There were 20 (23.52%) articles written by three authors, 15 (17.65%) by two authors, 13 (15.29%) by four authors, 13 (15.29%) by more than six authors, 10 (11.76%) by five authors, and seven (8.23%) by six authors as well as by a single author. Most of the articles were published in 2012, 12 in total, followed by 2007 with 11 and 2010 with 10.

Note that only 38 (44.7%) articles were written by authors affiliated with educational institutions (universities), followed by nine (10.59%) articles written jointly by authors affiliated with educational institutions and hospitals. In 31 (36.47%) articles, no data were found for this variable. The countries with the greatest scientific contributions were Brazil, with 16 papers (18.8%); the US, with 15 (17.6%); Spain, with six (7%); and the United Kingdom, with five (5.9%). When grouped by continent, America stands out with 37 (43.6%) articles, followed by Europe with 21 (24.7%).

In 98.82% of the articles, the population studied was represented by informal caregivers in their community or place of residence. The individuals with SMI were the subject of study in only 1.18% of the cases. Even when caregivers were the only population studied, as occurred in 57.64% of the papers, they were not the only subjects of study in this field. Both family caregivers and people with SMI appeared in 35.3% of the studies, whereas 3.53% of the articles focused on both family and formal caregivers. Finally, 2.35% of the articles analyzed focused on people with SMI along with both types of caregivers.

Regarding the type of study, 71 (83.54%) of them were primary, and seven (8.23%) were secondary. The remaining articles dealt with the validation of assessment instruments (7.06%) or provided no data on the type of study (1.17%). Regarding methodological design, 25 (29.42%) and 46 (54.12%) of the studies presented qualitative and quantitative analyses, respectively. The studies with qualitative methodology were classified as follows: three (12%) ethnographic, seven (28%) phenomenological, five (20%) grounded theory, one (4%) participatory action research, three (12%) biographical, and six (24%) unspecified. Of the studies using quantitative methodology, eight (17.39%) were randomized clinical trials, 31 (67.40%) were descriptive, three (6.52%) used cohorts, one (2.17%) was a case-control study, and three (6.52%) were quasi-experimental papers. 

For data collection, 38.14% of the studies used questionnaires; 37.11% used scales, and 12.37% used semistructured interviews. Only 4.12% used a DB for this purpose. The qualitative techniques used in the remaining studies accounted for no more than 4%, and 2.58% of the articles provided no data collection information. The most commonly used data treatments were univariate (28.57%) and bivariate (27.82%) statistical tests, whereas content analysis and multivariate analysis were used in 17.30 and 12.78% of the studies, respectively. A total of 9.02% of the articles provided no data.

## Discussion

Integrative reviews constitute a type of research that combines experimental and nonexperimental approaches to achieve a complete understanding of the phenomenon analyzed, integrating data from empirical and theoretical literature^(^ 21 ^)^. For the topic studied, this type of research provides not only expanded knowledge but also a state-of-the-art synthesis and the detection of knowledge gaps for future analyses.

Investigating the scientific literature related to home-based family caregivers of people with SMI requires a multidisciplinary approach because of the involvement of different areas and disciplines. Such an approach is implemented by consulting different databases. In the present study, consulting 14 multidisciplinary DBs allowed for the retrieval of the maximum number of articles. Additionally, by restricting the research scope to SMI within the family home, only one-tenth of the retrieved articles met the inclusion criteria (85 of 787 studies).

Focusing on home family care entails precise and specific DB searches and the use of search strategies and inclusion criteria^(^ 24 ^)^. In the present case, the DBs were JBI COnNECT and Cochrane Plus, both crucial to evidence-based clinical practice. Nonetheless, this study should not obstruct further investigation into other types of descriptive studies in different DBs^(^ 21 ^)^ that would advance the understanding of home-based family caregiving for people with SMI.

The 85 scientific articles retrieved were published in 61 different scientific journals. Such a breadth of publications reflects the importance of home-based care of people with SMI in both social and health disciplines. The meticulous study of these publications showed that more than one-third of the journals examined fell within the scope of nursing knowledge. This constitutes a significant finding because the search strategy did not include such key words as "*enfermería*" (DeCS) and "nursing" (MeSH) and was not limited to databases specific to the nursing discipline.

Of the 22 (100%) journals in the scientific field of nursing, 11 (50%) had an impact factor^(^ 25 ^)^. This fact shows that the home-based family care of people with SMI is a topic of interest to both new nursing researchers and nurses with a well-established research trajectory.

More than half of the journals in the nursing field were published on the American continent, followed by Europe and Australia. The main editor country was Brazil, followed by the United Kingdom and the US. A study by Juve Udina et al.^(^ 26 ^)^ on scientific literature in the field of nursing states that Anglo-Saxon countries are the world's largest producers of publications in this area (known as the Nightingale effect), and emerging countries, particularly China, Taiwan and Brazil, take second place (as an effect of economic development). These results differ from those of the present study, possibly because of the specificity of the topic researched. The aforementioned study^(26) ^examines the scientific literature of nursing in general, whereas the present review is limited to that concerning the home-based family care of people with SMI. In this sense, Brazil could be the main producer of articles, particularly because Brazil was implementing psychiatric reform during the development of this study. By contrast, the absence of publications on this topic in specific Asian countries, such as China and Taiwan, could point to a different rate of evolution in mental health reform or stem from the inclusion criteria used in the present review. 

There was a steady increase in the publication of articles during the period under investigation, presenting an upward trend from 2005 to 2012. Such an increase could be due to the visibility of family care and the evident need of joint work between health care professionals and informal caregivers in the field of mental health. Formal and informal collaborative work represents progress toward system sustainability. Most articles on family care were written by several authors, indicating that this topic has usually been examined by teams. Furthermore, university centers predominated in the publication of articles. However, collaboration between authors from clinical and teaching settings is becoming a reality. This pedagogical-clinical collaboration is essential for obtaining the best evidence and applying it to clinical practice. 

The majority of articles analyzed here are primary studies. These studies serve as a basis for future research, including secondary research, which facilitates data production for clinical practice. Research on the home-based family care of people with SMI mainly presented quantitative designs, relegating qualitative methodology to the background. In addition, there was a conspicuous lack of long-term studies (longitudinal studies) that follow-up on family care and explore its strengths and weaknesses to identify areas that require improvement in the future. Given this finding, the nature of nursing as a discipline should be reviewed in the light of potential contributions from the use of qualitative methodology. Providing care for individuals in a holistic way requires qualitative research capable of exploring the complexity and the sociocultural context of the individual^(^ 23 ^)^. For further understanding on experiences related to family care, close attention is required, and nursing professionals are fully trained for this purpose. Qualitative methodology has the potential to provide a better understanding of family care practice^(^ 27 ^)^. 

For subject matter, the most studied topic was work overload, particularly that of the family caregiver. The concern for this topic may result from changes internationally in the model of mental health care^(^ 11 ^)^, shifting from formal to informal care. In Spain, family caregivers provide 88% of care, whereas formal caregivers provide 12%^(^ 28 ^-^ 29 ^)^.

Analyzing the subjective perspective of family caregivers, the second most researched topic, allows health professionals to gain a better understanding of family care. As a result, this increased knowledge can be used in the development of guidelines and training specifications for family caregivers, whether novice or not, of people with SMI in the family home, thus applying research knowledge to clinical practice. Different authors^(^ 14 ^,^ 30 ^-^ 32 ^)^ have pointed to the importance of analyzing caregiver perception in evaluating the impact of family care and the positive aspects of tending a family member. Expanding the horizon of health care in nursing will bring a new perspective focused on the positive aspects and effects of home-based family care as well as on well-researched negative aspects, such as caregiver burnout^(^ 30 ^,^ 33 ^)^. Additionally, more studies are needed on burnout prevention and the maintenance of family caregivers' health^(^ 31 ^)^.

Finally, regarding resources, the current study demonstrated that articles analyzing the implementation of new technologies stand out in the mental health field.

One limitation of this study is that international variability in the meaning of the keyword home/*hogar* interfered with the selection of articles by introducing articles that did not correspond to the topic of study. Differences between mental health systems and their varied development in primary health care are additional limitations that were encountered^(^ 11 ^,^ 34 ^)^. Each country presents different degrees of development regarding health resources, despite the recommendations made by international agencies.

The varied social, political, economic, and developmental factors of health professions in each country should be considered as well. These factors may in turn influence the scientific literature. Finally, gray literature (doctoral theses, unpublished reports, etc) on the subject should also be analyzed.

## Conclusions

The databases with greater experience in the field hosted more articles on the topic of this study. Moreover, searching specific databases in education, social sciences, or psychology did not contribute articles to this review.

A wide range of journals was sensitive to the subject matter, and one-third of them belonged in the nursing field. This fact shows the interest of nursing researchers in home-based family care of people with SMI. This interest highlights the importance of care in nursing science, and both new and experienced researchers should focus scientific production on caregiving.

Primary research studies constituted much of the research analyzed. In accordance with the prevailing paradigm in health sciences, quantitative methodology carried greater weight in this review. 

Family overload was the topic most thoroughly studied; nonetheless, research related to subjective perspectives on family care and the professional caregiver-family caregiver relationship was represented in research regarding the home-based family care of those with SMI. A more exhaustive understanding of home-based family care of people with SMI-through increased research in all disciplines-can facilitate the documentation of progress and obstacles in family care as well as the redirection of resources to better meet the needs of the family caregiver of people with SMI. Nursing professionals and formal caregivers who already cater to informal caregivers or families-or those that will increasingly be obligated to do so by mental health reform-can apply this analysis in the development of their clinical practice.
